# Strontium Chloride: Can It Be a New Treatment Option for Ulcerative Colitis?

**DOI:** 10.1155/2014/530687

**Published:** 2014-06-18

**Authors:** Firdevs Topal, Ozlem Yonem, Nevin Tuzcu, Mehmet Tuzcu, Hilmi Ataseven, Melih Akyol

**Affiliations:** ^1^Gastroenterology Clinic, Ataturk Training and Research Hospital, 35965 Izmir, Turkey; ^2^Department of Gastroenterology, Faculty of Medicine, Cumhuriyet University, 58140 Sivas, Turkey; ^3^Cumhuriyet Universitesi Hastanesi, Gastroenteroloji B.D., 58140 Sivas, Turkey; ^4^Department of Pharmaceutical Microbiology, Faculty of Pharmacy, Cumhuriyet University, 58140 Sivas, Turkey; ^5^Department of Pathology, Faculty of Veterinary Medicine, Cumhuriyet University, 58140 Sivas, Turkey; ^6^Gastroenterology Clinic, Anadolu Hospital, 58070 Sivas, Turkey; ^7^Department of Dermatology, Faculty of Medicine, Cumhuriyet University, 58140 Sivas, Turkey

## Abstract

*Background/Aims*. Patients with ulcerative colitis still need effective therapy without major side effects. It has been found that strontium can suppress NF*κ*B activation induced by TNF-*α*. This opens a gate to a new anti-TNF agent which is cheap and can be given orally. We for the first time aimed to investigate the effect of strontium chloride (SrCl_2_) on inflammation in experimental colitis. *Methods*. Thirty female Wistar albino rats were divided into 5 groups each containing 6 rats. The rats in groups 1 and 2 served as the healthy control and colitis group, respectively. The rats in groups 3, 4, and 5 had colitis and received 40 mg/kg SrCl_2_, 160 mg/kg SrCl_2_, and 1 mg/kg prednisolone by oral gavage, respectively. The rats were sacrificed for histological evaluation and determination of serum neopterin, TNF-*α*, and IFN-*γ* levels. *Results*. The neopterin, TNF-*α* and IFN*γ* levels of group 2 was significantly higher than the other groups. The neopterin, TNF-*α*, and IFN-*γ* levels of controls and other treatment groups were comparable. There were a significant difference in macroscopic and microscopic healing between group 2 and other groups histologically. But there was not a significant difference within treatment receiving groups. *Conclusion*. SrCl_2_ had comparable therapeutic efficiency with prednisolone.

## 1. Introduction

Inflammatory bowel disease is a chronic intestinal inflammatory condition, the pathogenesis of which is complex and not fully understood. It includes mainly two diseases, namely, ulcerative colitis and Crohn's disease, that still need effective and lasting therapy without major side effects.

Available evidence suggests that an abnormal immune response against the microorganisms of the intestinal flora is responsible for the disease in genetically susceptible individuals [1]. Increases in the production of proinflammatory cytokines by both lymphocytes and macrophages have also been implicated in the pathogenesis of inflammatory bowel diseases [2]. NF-*κ*B activation is seen in mucosal biopsy specimens from patients with active Crohn's disease and ulcerative colitis. Treatment of patients with inflammatory bowel diseases with steroids decreases NF-*κ*B activity in biopsy specimens and reduces clinical symptoms. These results suggest that stimulation of the NF-*κ*B pathway may be involved in the enhanced inflammatory response associated with these diseases [3].

Strontium salts are used in routine clinical practice for treatment of osteoporosis, sensitive teeth, and bone pain related to bone metastasis [4–6]. Recently strontium was found to block TNF-*α* induced NF*κ*B signal transduction and in another article it was found to inhibit inflammatory mediators production by human monocytes [7–9]. All of these developments make strontium suitable for use in inflammatory diseases. For this reason for the first time in the literature we investigated the effect of SrCl_2_ on inflammation in experimental colitis and compared it with a novel therapeutic agent prednisolone.

## 2. Methods

### 2.1. Ethics

The present study was approved by the Animal Ethics Committee of Cumhuriyet University in accordance with the Standards for the Care and Use of Laboratory Animals.

### 2.2. Animals

Thirty female Wistar albino rats each weighing about 180–200 grams were included in the study. They were maintained under standard conditions (12 h light/dark cycle; 25 ± 3°C, 45–65% humidity) and had free access to standard rat feed and water* ad libitum*. The experiments were performed during the light portion between 08:00 and 12:00 a.m. to avoid circadian influences.

### 2.3. Serum Proinflammatory Cytokines

Serum levels of neopterin (Rat Neopterin ELISA- Cusabio,), TNF-*α* (Rat TNF-*α* Platinum ELISA- eBioscience), and IFN-*γ* (Rat IFN-*γ* Platinum ELISA- eBioscience) were determined in the groups by ELISA method. Thermo Multiskan device was used for ELISA applications.

### 2.4. Induction of Colitis

Experimental ulceration in colon tissue was done according to the method described by Mousavizadeh et al [10] with slight modification. Under light ether anesthesia rats were administered 2 mL of 4% acetic acid solution in ethanol by transrectally using a (2.7 mm) soft pediatric catheter. After acetic acid administration, rats were holed horizontally for 2 minutes to prevent acetic acid leakage. Then the animals were divided into 5 groups each containing 6 rats. The rats in group 1 served as the healthy control group and we did not induce colitis in them. We induced colitis in groups 2–5. The rats in group 2 served as the colitis group which received no medication; the rats in group 3 had colitis and received 40 mg/kg strontium chloride; the rats in group 4 had also colitis but received 160 mg/kg strontium chloride; the rats having colitis in group 5 received 1 mg/kg prednisolone. All these treatments were given for seven days orally by using oral gavage.

Seven days after the induction of colitis, rats in all groups including groups 1–5 were sacrificed by cervical subluxation, blood was obtained by retroorbital puncture for biochemical estimation, and the colon was removed and postfixed. Sections of the colon were stained for hematoxylin-eosin and evaluated microscopically and scored for inflammation.

### 2.5. Histology

#### 2.5.1. Macroscopy

After sacrificing animals, the colon was carefully dissected from anus till rectum and placed in saline. The colon was opened longitudinally over the mesenteric border and washed gently in saline. It was placed on a rubber mat with the mucosal side up and evaluated macroscopically. Macroscopic changes in colonic mucosa were scored from 0 to 3 with score 0 referred to preserved mucosa with normal appearance, score 1 to edema and congestion, score 2 to ulceration and hemorrhage, and finally score 3 to abundant multiple ulcers and thinned bowel wall.

#### 2.5.2. Microscopy

After macroscopic evaluation, the colon was then placed with the serosal side up and dissected. Serial sections of 6 mm were obtained and fixed with %10 buffered formaldehyde. They were embedded in paraffin blocks. Sections of 5 *µ*m were prepared from these blocks and stained with hematoxylin and eosin and scored for inflammation. Severity and abundance of edema, necrosis, inflammatory infiltration, apoptosis, thrombosis-vascular congestion, and shedding of epithelial cells were considered during microscopic scoring. Histopathological evaluation of the slides was performed by one pathologist who was blinded about the groups. Microscopic changes were scored from 0 to 3 with score 0 referred to no swelling in epithelial cells, normal crypt appearance, sporadic monocyte infiltration, either sporadic or none neutrophilic infiltration; score 1 to moderate blurred swelling in epithelial cells, sporadic polymorphonuclear leukocyte infiltration in crypts, mild monocyte, and neutrophil infiltration in submucosa; score 2 to shedding of epithelial cells and flatness of mucosa, shedding of crypt epithelium, moderate monocytic-neutrophilic infiltration of submucosa; and score 3 to marked epithelial ulcers, crypt abscesses, and marked monocytic and neutrophilic infiltration of submucosa.

### 2.6. Statistical Analysis

Data were analyzed by using SPSS 15.0 package software. As the assumptions of parametric tests could not be fulfilled, Kruskal Wallis, Mann-Whitney U test, and Chi-square tests were used for statistical evaluation. A *P* value of <0.05 was considered to be significant.

## 3. Results

### 3.1. Serum Proinflammatory Cytokines

We compared the mean serum neopterin, TNF-*α*, and interferon-*γ* levels of groups after 7 days of treatment.

**Figure 1:**
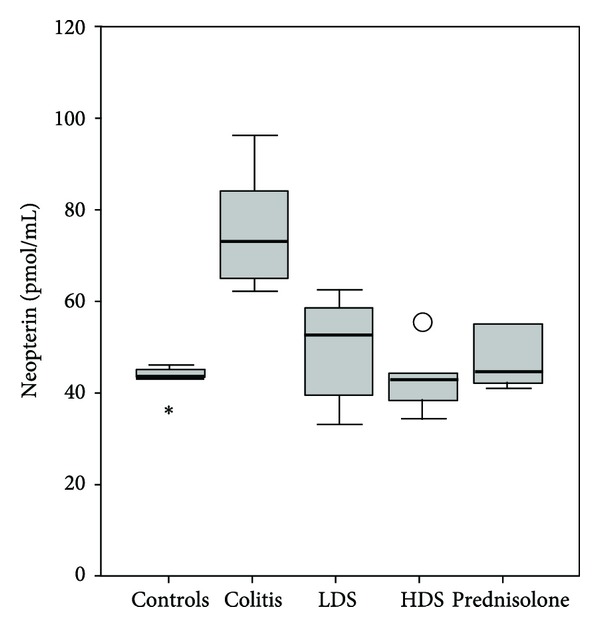
Comparison of neopterin levels among groups. The horizontal lines in the middle of each box indicate the median, while the top and bottom borders of the box mark the 25th and 75th percentiles, respectively. The whiskers above and below the box mark the maximum and minimum neopterin levels. Open circles indicate outliers. Asterisks represent extreme cases.

**Figure 2:**
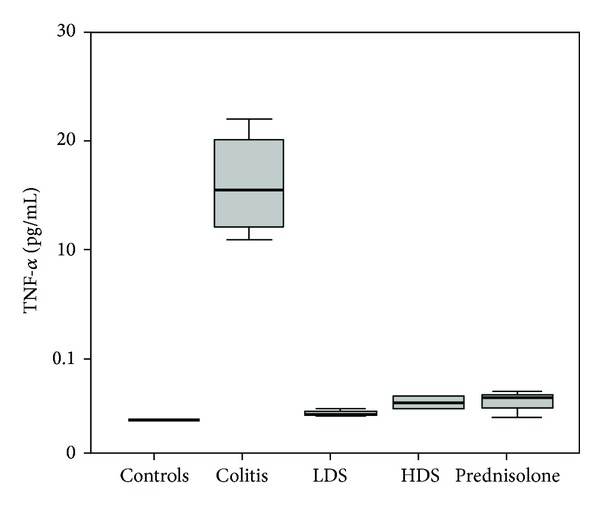
Comparison of TNF-alpha levels among groups. The horizontal lines in the middle of each box indicate the median, while the top and bottom borders of the box mark the 25th and 75th percentiles, respectively. The whiskers above and below the box mark the maximum and minimum TNF-alpha levels.

**Figure 3:**
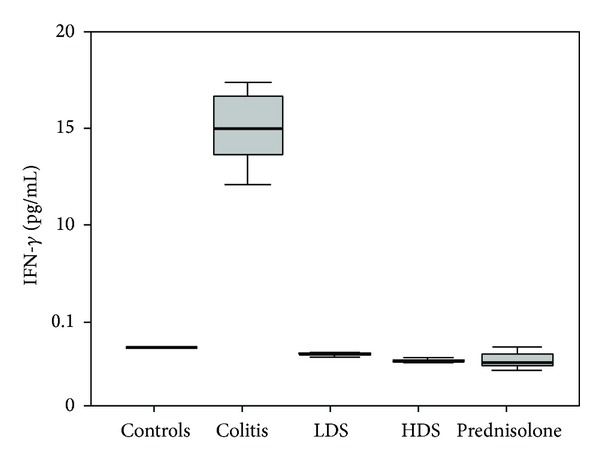
Comparison of IFN-gamma levels among groups. The horizontal lines in the middle of each box indicate the median, while the top and bottom borders of the box mark the 25th and 75th percentiles, respectively. The whiskers above and below the box mark the maximum and minimum IFN-gamma levels.

The neopterin, TNF-*α*, and interferon-*γ* levels of colitis group (group 2) were significantly higher than those of the group 1 (healthy control), group 3 (40 mg/kg: low dose strontium), group 4 (160 mg/kg: high dose strontium), and group 5 (prednisolone) (*P* < 0.05). The neopterin, TNF-*α*, and interferon-*γ* levels of controls, low dose strontium, high dose strontium, and prednisolone groups were comparable (*P* > 0.05) (Figures 1, 2, and 3).

### 3.2. Histopathological Studies

**Figure 4:**
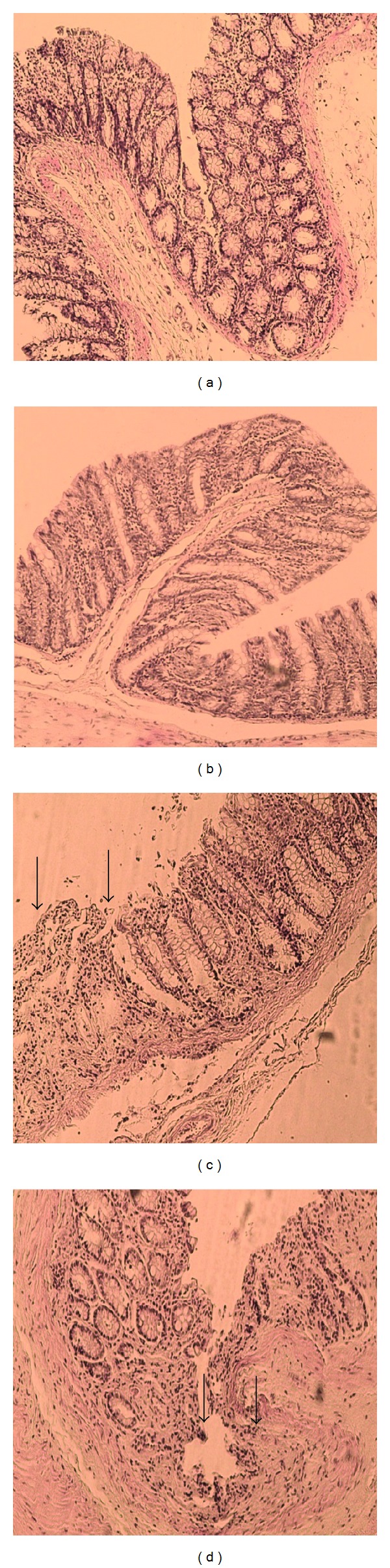
Histopathological changes in the colon of experimental rats. (a) Normal intact mucosa from normal colon, hematoxylin/eosin ×260. (b) Appearance of colon in low-dose strontium group, hematoxylin/eosin ×400. (c) Arrows show healed ulcer in the low dose strontium group, hematoxylin/eosin ×260. (d) Appearance of colon in the ulcerative colitis group; arrows show ulcers, hematoxylin/eosin ×260.

There was a significant difference by means of both macroscopic and microscopic scores between group 2 (The colitis group) and other four groups (*P* < 0.05). But there was no significant difference within treatment receiving groups (groups 3, 4 and 5) when compared in pairs (*P* > 0.05).  Table 1 shows the macroscopic and microscopic scores of all rats involved in this study.  Figure 4 shows the histopathological changes in the colon of experimental rats.

## 4. Discussion

We have found in our study for the first time in the literature that strontium chloride had comparable therapeutic efficiency when compared with prednisolone in a rat ulcerative colitis model. We investigated the effect of strontium at two doses, 40 mg/kg and 160 mg/kg, but did not find a difference between these two doses by means of histological and biochemical healing.

The immune system plays a dominant role in inflammatory bowel disease. During active inflammation, Th0 cells differentiate into T helper cell type (Th)1, Th17, and/or Th2 cells, upon the action of interleukin- (IL-) 12/IL-18, IL-6/TGF-*β*, or IL-4, respectively. Th1 cells, in turn, produce vast amounts of interferon- (IFN-) *γ* and tumor necrosis factor- (TNF-) *α*. IFN-*γ* induces the production of further TNF-*α* by intestinal activated macrophages and triggers apoptosis of epithelial cells. TNF-*α* promotes the differentiation of lamina propria stromal cells into activated myofibroblasts, which start producing high quantities of tissue-degrading matrix metalloproteinases (MMPs). MMPs degrade the extracellular matrix and the basement membrane, thus inducing enterocyte apoptosis [1].

Neopterin, a pyrazino[2,3-d]-pyrimidine compound, is a metabolite of cyclic guanosine monophosphate that is released by activated dendritic cells and macrophages after induction by *γ* interferon [11]. Neopterin concentration increase in the blood reflects the activation of cellular immunity and it has been suggested that it is an excellent marker for the monocyte/macrophage axis in some clinical situations [12]. We have found the serum TNF-*α*, IFN-*γ*, and neopterin values to be significantly increased in our rat colitis model.

Strontium salts may also directly act on nonneuronal cells such as keratinocytes or immunoregulatory inflammatory cells. Recent reports demonstrate that strontium salts can suppress keratinocyte derived tumor necrosis factor-alpha (TNF-*α*), interleukin-1 *α* (IL-1 *α*), and IL-6 in in vitro cultures [13]. It has also been shown that strontium can suppress NF*κ*B activation induced by TNF-*α* and can inhibit inflammatory mediators production by human monocytes [7–9]. Considering all these effects of strontium, we hypothesized that it can be beneficial in inflammatory bowel disease. Macroscopically in histology all of our ulcerative colitis group rats received the score 3 which meant abundant multiple ulcers and thinned bowel wall; microscopically again all of our ulcerative colitis rats received the score 3 which meant marked epithelial ulcers, crypt abscesses, and marked monocytic and neutrophilic infiltration of submucosa. We observed the healing effect of strontium both macroscopically and microscopically that is comparable with prednisolone.

Strontium has been used to treat patients with osteoporosis or other disorders of bone mineralization. Treatment has involved administration of “low doses” of strontium salts (usually the lactate, gluconate, carbonate, or ranelate) over several years. No adverse side-effects were reported in studies on osteoporosis patients [14]. Beneficial effects on bone mineralization (a 17% increase in mineral bone volume and a 70% increase in the number of bone-forming sites) and no adverse effect on the hydroxyapatite mineral particle size were observed in rats at a dose of 168 mg/kg [15]. And Kroes et al. investigated short-term toxicity of strontium chloride in rats at doses of 2.6, 10, 40, and 180 mg/kg [16]. We used strontium at two doses, namely, 40 mg/kg and 160 mg/kg, and found no significant difference about the therapeutic efficiency of these two doses. New rat studies investigating the minimum effective strontium dose on colitis in rats can be planned.

In conclusion, we have found in our study that strontium chloride has comparable therapeutic efficiency with prednisolone in a rat ulcerative colitis model. We have found the dose of 40 mg/kg to be effective in suppressing inflammation in rats; however, new studies investigating the efficiency of lower doses can be planned. Our findings have important implications in that a new way to an alternative therapy for nonresponder patients is opened and a hope for osteoporotic ulcerative colitis patients emerged with one medication for two diseases. However, further studies in different phases are needed to confirm our results.

## Figures and Tables

**Table 1:** Macroscopic and microscopic scores of the each 6 rats in the five groups.

	Group 1	Group 2	Group 3	Group 4	Group 5
Macroscopic∗ scores	0	3	2	1	1
	0	3	1	1	2
	0	3	2	2	1
	0	3	0	2	2
	0	3	2	2	2
	0	3	0	1	2
Median ± SEM	0	3	1.50 ± 0.40	1.50 ± 0.22	2.00 ± 0.21

Microscopic∗ scores	0	3	1	2	1
	0	3	1	1	2
	0	3	2	2	1
	0	2	1	1	1
	0	3	1	1	0
	0	3	0	2	2
Median ± SEM	0	3	1.00 ± 0.25	1.50 ± 0.22	1.00 ± 0.30

*There was a significant difference by means of both macroscopic and microscopic scores between group 2 (The colitis group) and other four groups (*P* < 0.05). But there was no significant difference within treatment receiving groups (groups 3, 4, and 5) when compared in pairs (*P* > 0.05).
